# Risk factor analysis for major mediastinal vessel invasion in thymic epithelial tumors based on multi-slice CT Imaging

**DOI:** 10.3389/fonc.2023.1239419

**Published:** 2023-09-11

**Authors:** Yu-Hui Ma, Jie Zhang, Wei-Qiang Yan, Jiang-Tao Lan, Xiu-Long Feng, Shu-Mei Wang, Guang Yang, Yu-Chuan Hu, Guang-Bin Cui

**Affiliations:** ^1^ Department of Radiology, Tangdu Hospital, Air Force Medical University (Fourth Military Medical University), Xi’an, Shaanxi, China; ^2^ Functional and Molecular Imaging Key Lab of Shaanxi Province, Xi’an, Shaanxi, China; ^3^ Department of Pathology, Tangdu Hospital, Air Force Medical University (Fourth Military Medical University), Xi’an, Shaanxi, China; ^4^ Department of Thoracic Surgery, Tangdu Hospital, Air Force Medical University (Fourth Military Medical University), Xi’an, Shaanxi, China

**Keywords:** thymoma, thymic carcinoma, vascular invasion, aorta, vena cava, superior, logistic regression model

## Abstract

**Objective:**

To explore the characteristics and risk factors for major mediastinal vessel invasion in different risk grades of thymic epithelial tumors (TETs) based on computed tomography (CT) imaging, and to develop prediction models of major mediastinal artery and vein invasion.

**Methods:**

One hundred and twenty-two TET patients confirmed by histopathological analysis who underwent thorax CT were enrolled in this study. Clinical and CT data were retrospectively reviewed for these patients. According to the abutment degree between the tumor and major mediastinal vessels, the arterial invasion was divided into grade I, II, and III (< 25%, 25 – 49%, and ≥ 50%, respectively); the venous invasion was divided into grade I and II (< 50% and ≥ 50%). The degree of vessel invasion was compared among different defined subtypes or stages of TETs using the chi-square tests. The risk factors associated with TET vascular invasion were identified using multivariate logistic regression analysis.

**Results:**

Based on logistic regression analysis, male patients (β = 1.549; odds ratio, 4.824) and the pericardium or pleural invasion (β = 2.209; odds ratio, 9.110) were independent predictors of 25% artery invasion, and the midline location (β = 2.504; odds ratio, 12.234) and mediastinal lymphadenopathy (β = 2.490; odds ratio, 12.06) were independent predictors of 50% artery invasion. As for 50% venous invasion, the risk factors include midline location (β = 2.303; odds ratio, 10.0), maximum tumor diameter larger than 5.9 cm (β = 4.038; odds ratio, 56.736), and pericardial or pleural effusion (β = 1.460; odds ratio, 4.306). The multivariate logistic model obtained relatively high predicting efficacy, and the area under the curve (AUC), sensitivity, and specificity were 0.944, 84.6%, and 91.7% for predicting 50% artery invasion, and 0.913, 81.8%, and 86.0% for 50% venous invasion in TET patients, respectively.

**Conclusion:**

Several CT features can be used as independent predictors of ≥50% artery or venous invasion. A multivariate logistic regression model based on CT features is helpful in predicting the vascular invasion grades in patients with TET.

## Introduction

Thymic epithelial tumor (TET) is the most common tumor in the anterior mediastinum, with an overall incidence of approximately 0.30/100,000 (1, 2). Histological types and complete surgical resection are two leading prognostic indicators evaluated by the world health organization (WHO) classification and Masaoka-Koga (MK) staging system, respectively (3, 4). TET includes thymoma, thymic carcinoma, and thymic neuroendocrine tumor, among which thymoma is subdivided into type A, AB, and B1 (low-risk thymoma), B2, and B3 (high-risk thymoma) (3, 5). MK or TNM staging divides TET into stages I, II (early stage), III, and IV (advanced stage) according to the adjacent structure infiltration and lymphatic/hematological spread (6–9). The histological types and stages are closely related to the treatment plan and prognosis of TETs (10).

The latest national comprehensive cancer network (NCCN) clinical practice guidelines for thymoma and thymic carcinoma indicate that early-stage lesions can be excised by direct surgery, while locally advanced cases need to be treated according to their resectability (8). The resectability of TETs is mainly affected by the degree of tumor invasion to adjacent large vessels (11, 12). The vascular abutment on computed tomography (CT) images is closely related to pathological vascular invasion. The abutment degree of adjacent vessels evaluated by CT imaging was an independent predictor of incomplete resection in TET patients (11, 12). Although innominate or superior vena cava (SVC) vessels can still be resected and reconstructed if necessary, infiltration of the arterial systems usually limits macroscopically complete resection of TETs (13). Therefore, it is critical to accurately identify the degree of vascular abutment or invasion for determining the optimal surgical approach and therapeutic plans before treatment.

According to previous research reports, CT features related to incomplete surgical resection include that the abutment of the adjacent vessel is larger than or equal to 50% in thymoma (>25% in thymic carcinoma) (11–13). In addition, measuring the interface length between the primary tumor and adjacent structures may be a simple, noninvasive method for evaluating vascular invasion (14). Although contrast-enhanced CT can provide direct information about the type, invasive depth, and endovascular status of the infiltrated vessels, it remains difficult for current imaging methods to accurately quantify the degree of vascular invasion adjacent to the tumor (11, 12, 15). Therefore, combining direct and indirect signs of vascular invasion may help improve the efficacy of predicting vascular invasion in TETs. However, to our knowledge, no previous study has focused on the indirect information of vascular invasion, namely risk factors for major mediastinal vessel invasion in TETs.

In this study, we intended to explore the characteristics and risk factors of major mediastinal vascular invasion among different histologic types, MK and TNM stages using multi-slice CT, and develop a logistic regression model incorporating the clinical and CT information for predicting the degree of vascular invasion in TETs.

## Materials and methods

### Patients

Ethical approval was obtained from the local Ethics Committee for this retrospective study, and informed consent was waived. This study was conducted in accordance with the Declaration of Helsinki.

**Figure 1:**
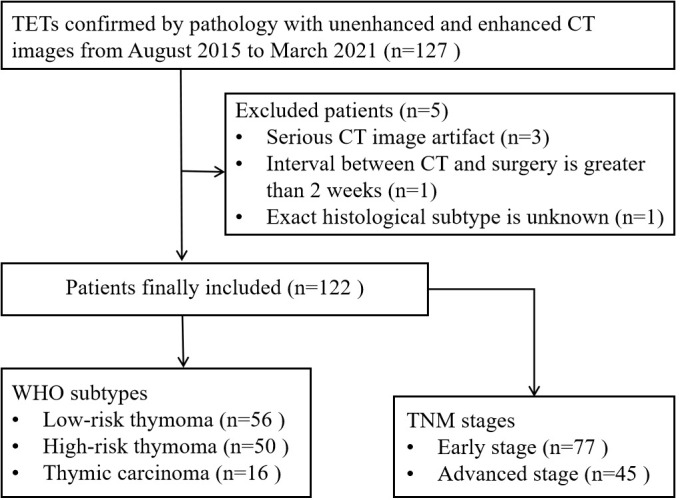
Flow diagram of patient selection. *TETs*, thymic epithelial tumors.

From August 2015 to March 2021, 127 patients with TET underwent thorax CT examinations were included according to the following criteria: (a) TETs confirmed by pathology; (b) both non-contrast and contrast-enhanced CT images were available; (c) lesions larger than 1 cm in diameter based on the longest diameter; (d) the patient did not undergo biopsy or any treatment before CT examinations. In addition, a total of 5 cases were excluded according to the following exclusion criteria: (a) severe CT image artifact (n=3); (b) interval between CT and surgery was greater than two weeks (n=1); (c) exact histological subtype was unknown based on the analysis of puncture biopsy specimen (n=1). Thus, the final study group comprised 122 patients (67 male, 55 female; mean age, 52 years; age range, 18-78 years) (**Figure 1**). Demographic and clinical data were retrieved from electronic medical records.

### Thorax CT Protocol

All thorax CT examinations were performed on 128-row CT scanner (Somatom Definition Flash, Siemens Healthcare, Forchheim, Germany), 256-row CT (Brilliance iCT, Philips Healthcare, Cleveland, USA), or 64-row CT (GE-Medical-Systems, Milwaukee, USA). Before the scan, the patient was instructed in breathing training. First, a non-enhanced thorax CT (210 mAs, 120 kV; slice thickness and interval, 5.0 mm) was performed. CT images were collected during a breath-holding period, and the scanning range was from the thoracic entrance to the costophrenic angle level in the cranio-caudal direction. Secondly, contrast medium was administered using a dual-head pump injector. A volume of 60-100 ml (1.2 ml/kg of body weight) iodixanol injection 320 (HengRui, JiangSu, China) was injected in the forearm vein at a flow rate of 3 ml/sec using a 20-G needle followed by a saline flush of 30 ml at the same rate. After intravenous injection of the contrast medium, the arterial and venous phase scan were performed, respectively. The scan parameters were as same as those of non-enhanced CT. The original data obtained from the scanning were reconstructed into axial, coronal, and sagittal images and then transmitted to the dedicated workstation.

### Image analysis

CT features of the tumors were analyzed individually by two experienced radiologists (Y.-H.M. and W.-Q.Y., with 7 and 12 years of experience in chest imaging, respectively) who knew the patient had TETs but were blinded to the histological subtypes of the tumors. The disagreements between the two readers were resolved by negotiation with another senior radiologist (Y.-C.H., with 18 years of experience in chest imaging) until a consensus was reached.

Evaluation of tumor conventional CT features refers to the standard report terms for thymoma defined by the International Thymic Malignancy Interest Group (ITMIG) (14). The recorded CT features include tumor location (off-midline and midline), maximum diameter, contour (smooth and lobulated), homogeneity (almost homogenous and heterogeneous), enhancement degree, infiltration of adjacent pericardium or pleura, pericardiac or pleural effusion, and lymphadenopathy (short-axis diameter >10 mm). The maximum diameter of the tumor was measured at the level where the tumor appeared largest on the cross-sectional image. The enhancement degree was divided into mild to moderate [net enhanced value ≤ 40 Hounsfield Unit(HU)] and strong (net enhanced value > 40 HU) (16). Lung invasion was considered based on signs: a multilobular tumor convex to the lung with adjacent lung abnormalities, or deep lobulation at the tumor-lung interface (17, 18). The pericardiac/pleural invasion was considered when the space between the tumor and the pericardiac/pleura disappeared, with pericardiac/pleura thickening and/or cavity effusion (17).

**Figure 2:**
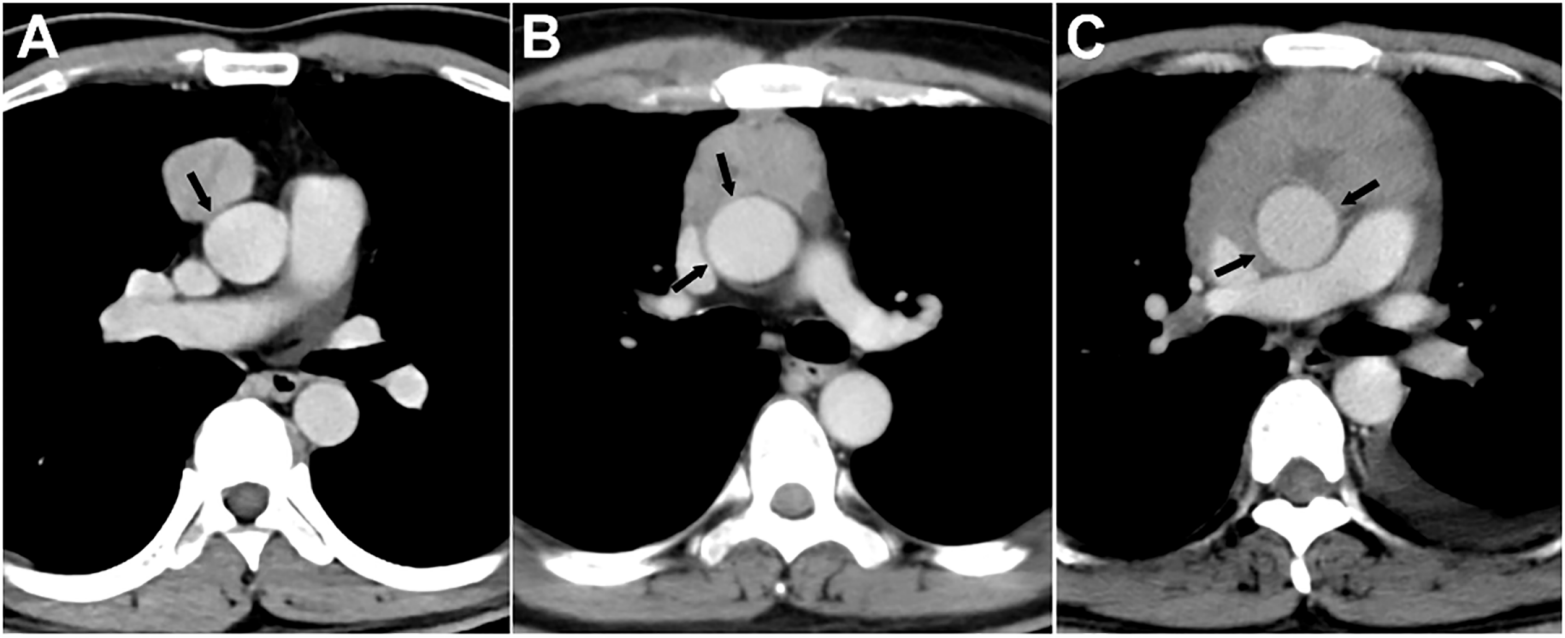
Grading of major mediastinal artery invasion on contrast-enhanced CT images. **(A)** Grade I, <25%. A 42-year-old man with type AB thymoma. Tumor abutment degree with adjacent ascending aorta is less than 25% (arrow) on axial venous phase enhanced CT image. **(B)** Grade II, 25%-49%. A 49-year-old man with thymic squamous cell carcinoma. Tumor abutment degree with adjacent ascending aorta is more than 25% but less than 50% (arrow) on axial venous phase enhanced CT image. **(C)** Grade III, ≥50%. A 40-year-old man with type B3 thymoma. Tumor abutment degree with adjacent ascending aorta is more than 50% (arrow) on axial venous phase enhanced CT image.

**Figure 3:**
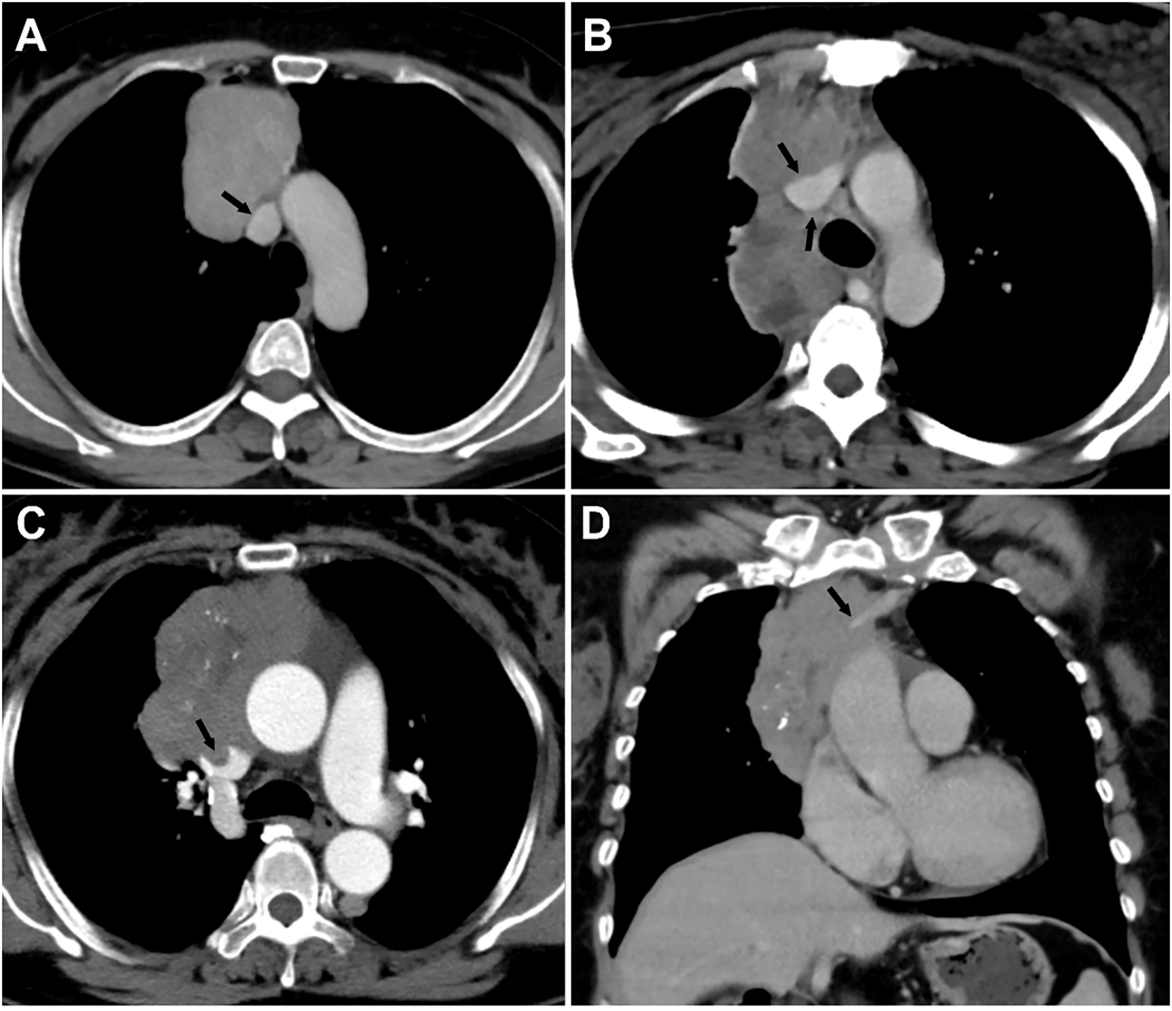
Grading of major mediastinal vein invasion on contrast-enhanced CT images. **(A)** Grade I, <50%. A 49-year-old woman with type AB thymoma. Tumor abutment degree with adjacent superior vena cava (SVC) is less than 50% (arrow) on axial venous phase enhanced CT image. **(B)** Grade II, ≥50%. A 30-year-old woman with type B3 thymoma. Tumor abutment degree with adjacent SVC is more than 50% (arrow) on axial venous phase enhanced CT image. **(C, D)** Grade II, ≥50%, with a filling defect in the SVC and occlusion of the left innominate vein. A 59-year-old woman with type B2 thymoma. Tumor abutment degree with adjacent SVC is more than 50% with filling defect in the SVC (arrow) on axial enhanced CT image **(C)**. Coronal venous phase enhanced CT image **(D)** reveals the occlusion of the left innominate vein (arrow).

The evaluated major mediastinal vessels included the aorta, branches of the aortic arch, pulmonary trunk, left and right pulmonary artery, SVC, innominate vein, and hilar pulmonary veins. According to the extent of tumor abutment with the adjacent vessel, the arterial invasion was divided into the following three grades: < 25%, 25-49%, and ≥ 50% (**Figure 2**); the venous invasion was divided into two grades: < 50% and ≥ 50% (**Figure 3**).

### Pathologic diagnosis

The final diagnosis was determined by surgical or puncture biopsy specimen and confirmed with histopathological examination. Based on the criteria of the WHO histological classification and Jeong simplification classification (5, 19), TET was divided into three subgroups: low risk thymoma (types A, AB, and B1), high risk thymoma (types B2 and B3) and thymic carcinoma. TETs were divided into stages I-IV according to the MK or TNM staging system (7, 9, 20).

### Statistical analysis

The Pearson chi-square test or Fisher’s exact test was used to analyze categorical variables among defined groups. The Bonferroni method was used for pairwise comparison between groups. Multivariate logistic regression analysis was used to identify the risk factors associated with TET vascular invasion. Cohen’s kappa coefficient with a 95% confidence interval was adopted to assess the inter-reader agreement level in evaluating the degree of tumors abutment of adjacent vessels circumference; kappa values were interpreted as follows: poor, 0–0.2; fair, 0.21–0.4; moderate, 0.41–0.6; good, 0.61–0.8; and excellent, 0.81–1.0. Using the clinical and CT features as independent variables, and defined group (vessel invasion grade) as dependent variables, binary logistic regression analysis was conducted to establish a regression model. The receiver operating characteristic (ROC) curve analysis was performed for each statistically significant feature, and the predicted probability value of the regression model, and the area under the curve (AUC), sensitivity, and specificity were obtained. All statistical analyses above were performed with IBM SPSS 26 software (IBM Corp). Statistical significance was accepted as P < 0.05.

## Results

### Patients’ characteristics

The demographic characteristics of the patients are summarized in **Table 1**. A total of 122 patients (67 males, 55 females) with a mean age of 52 years were retrospectively entered into the study. Among these patients, 38 patients (31.1%) had been diagnosed with myasthenia gravis, and other clinical features included chest pain (17.2%, 21 of 122), respiratory symptoms (13.1%, 16 of 122), others (13.9%, 17 of 122), and no symptom in 30 patients (24.6%).

**Table 1:** Clinical and demographic characteristics of 122 patients with thymic epithelial tumors.

Patients’ characteristics	value
Age (years, Mean ± SD)	51.5 ± 11.7
Gender, n (%)	
Male	67 (54.9)
Female	55 (45.1)
Symptoms, n (%)	
Myasthenia gravis	38 (31.1)
Chest pain	21 (17.2)
Respiratory symptoms	16 (13.1)
Other	17 (13.9)
No symptom	30 (24.6)
Method for obtaining pathologic results, n (%)	
VATS	74 (60.7)
Thoracotomy	35 (28.7)
Biopsy	13 (10.6)
Masaoka-Koga stage, n (%)	
I	9 (7.4)
II	62(50.8)
III	22 (18.0)
IV	29 (23.8)
TNM stage, n (%)	
I	72 (59.0)
II	5 (4.1)
III	16 (13.1)
IV	29 (23.8)
Histological type, n (%)	
Thymoma	106 (86.9)
Type A	13 (10.7)
Type AB	33 (27.0)
Type B1	10 (8.2)
Type B2	32 (26.2)
Type B3	18 (14.8)
Thymic carcinomas	16 (13.1)
Squamous cell carcinoma	13 (10.7)
Thymic neuroendocrine tumor	3 (2.4)

SD, standard deviation. VATS, video-assisted thoracoscopic surgery.

The pathological results were proved on the specimen, obtained by thoracotomy in 35 patients (28.7%), thoracoscopy in 74 patients (60.7%), and percutaneous biopsy in 13 patients (10.6%). After surgery and pathological analysis, 77 patients (63.1%) were found to be in TNM stage I or II, 45 (36.9%) in stage III or IV, and 71 patients (58.2%) in MK stage I or II, 51 (41.8%) in stage III or IV. Thirteen masses did not undergo surgery, and tumor stage was evaluated by puncture biopsy and imaging. Histological analysis revealed 106 (86.9%) thymomas (13 of type A, 33 of type AB, 10 of type B1, 32 of type B2, and 18 of type B3), 13 thymic squamous cell carcinoma, and 3 thymic neuroendocrine tumors (**Table 1** and **Supplemental Table 1**).

### Comparison of major mediastinal vascular abutment in different types and stages of TETs

**Figure 4:**
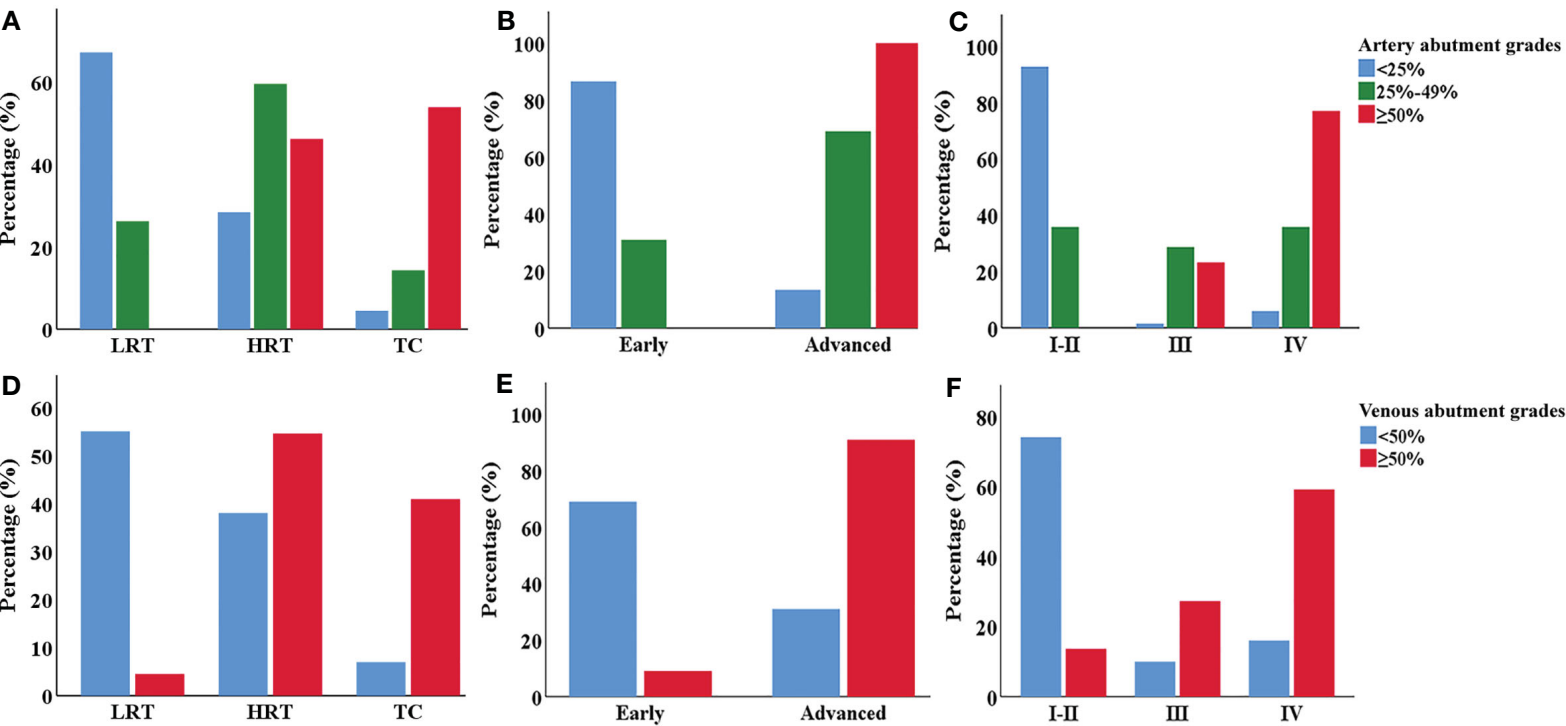
The clustered bar graph of major mediastinal vessel abutment grades in different types and stages of TETs. **(A–C)** Comparison of the artery vessel abutment grades among different types **(A)**, MK **(B)** and TNM stages **(C)** of TETs. **(D–F)** Comparison of the venous vessel abutment grades among different types **(D)**, MK **(E)** and TNM stages **(F)** of TETs. *LRT*, low-risk thymoma. *HRT*, high-risk thymoma. *TC*, thymic carcinoma.

Comparisons of vascular abutment among groups with different types and stages of TETs are shown in **Table 2** and **Figure 4**. Overall, there were significant differences in the abutment rate of major mediastinal vessels among low-, high-risk thymomas, and thymic carcinomas, and between early and advanced stages of TETs (all P < 0.01).

**Table 2:** Comparison of major mediastinal vascular invasion in different types and stages of thymic epithelial tumor.

Variables	Simplified WHO histological types			*P*	Masaoka-koga stages		*P*	TNM stages			*P*
	LRT (n = 56)	HRT (n = 50)	TC (n = 16)		Early (n = 71)	Advanced (n = 51)		I-II (n = 77)	III (n = 16)	IV (n = 29)	
Aorta and its branches, n (%)				< 0.001* ^a,b^			< 0.001^*^				< 0.001* ^a,b^
≥ 50%	0 (0.0)	6 (12.0)	5 (31.3)		0 (0.0)	11 (21.6)		0 (0.0)	2 (12.5)	9 (31.0)	
< 50%	56 (100.0)	44 (88.0)	11 (68.8)		71 (100.0)	40 (78.4)		77 (100.0)	14 (87.5)	20 (69.0)	
Pulmonary artery and its branches, n (%)				< 0.001^* b^			0.001^*^				< 0.001* ^a,b^
≥ 50%	0 (0.0)	3 (6.0)	5 (31.3)		0 (0.0)	8 (15.7)		0 (0.0)	2 (12.5)	6 (20.7)	
< 50%	56 (100.0)	47 (94.0)	11 (68.8)		71 (100.0)	43 (84.3)		77 (100.0)	14 (87.5)	23 (79.3)	
SVC and innominate vein, n (%)				< 0.001^# a,b,c^			< 0.001^#^				< 0.001* ^a,b^
≥ 50%	1 (1.8)	12 (24.0)	9 (56.3)		2 (2.8)	20 (39.2)		3 (3.9)	6 (37.5)	13 (44.8)	
< 50%	55 (98.2)	38 (76.0)	7 (43.8)		69 (97.2)	31 (60.8)		74 (96.1)	10 (62.5)	16 (55.2)	
Pulmonary vein, n (%)				0.007 * ^b^			0.002^*^				< 0.001* ^b^
≥ 50%	0 (0.0)	4 (8.0)	3 (18.8)		0 (0.0)	7 (13.7)		0 (0.0)	1 (6.3)	6 (20.7)	
< 50%	56 (100.0)	46 (92.0)	13 (81.3)		71 (100.0)	44(86.3)		77 (100.0)	15 (93.8)	23 (79.3)	

Comparisons among groups using ^#^Pearson chi-square tests or ^*^ Fisher’s exact tests. p < 0.05 indicates a statistically significant difference among all groups, and adjusted p < 0.0167 (0.05/3, Bonferroni method) indicated a significant difference between the two groups. ^a^ Represents significant differences between LRT and HRT groups, or between TNM I-II and III. ^b^ Represents significant differences between LRT and TC groups, or between TNM I-II and IV. ^c^ Represents significant differences between HRT and TC groups. LRT, low-risk thymoma. HRT, high-risk thymoma. TC, thymic carcinoma. SVC, superior vena cava.

For the aorta and its branches, 6 cases (12%) of high-risk thymoma and 5 cases (31.3%) of thymic carcinoma, or 11 cases of advanced TETs, had vascular abutment ≥ 50% of the vascular circumference. For the pulmonary artery and its branches, 3 cases (6%) of high-risk thymoma and 5 cases (31.3%) of thymic carcinoma, or 8 cases of advanced TETs, had vascular abutment ≥ 50% of the vascular circumference. As for SVC or innominate vein, the abutment rate differed among different types or stages (P < 0.001), with the highest abutment rate being thymic carcinoma (9/16, 56.3%), followed by advanced TETs, and high-risk thymoma (12/50, 24.0%). The abutment rate of the pulmonary veins was relatively low, with vascular abutment ≥ 50% of the vascular circumference in only 4 cases (8%) of high-risk thymoma and 3 cases (18.8%) of thymic carcinoma, or 7 cases of advanced TETs.

In addition, whether it is an artery or a vein, ≥ 50% of vascular abutment was mainly seen in stages III and IV, with significant statistical significance compared to stage I and II (P < 0.0167) based on TNM stages, except for pulmonary vein invasion among stage I-II and stages III (P > 0.0167); However, there was no significant statistical significance between the III and IV stages of vascular abutment with ≥ 50% (P > 0.0167).

### Association between clinical and CT features and the grades of major mediastinal vascular invasion in TETs

The association between clinical and CT features and artery invasion grades of TETs is shown in **Table 3**. Age, myasthenia gravis, and tumor location were not statistically different among the three groups (P > 0.05). It showed a higher rate of arterial invasion in male patients and tumors with a maximum diameter larger than 5.9 cm, lobulated contours, heterogeneous density, mild to moderate degree of enhancement, pericardial or pleural invasion, adjacent lung invasion, pericardial or pleural effusion, and mediastinal lymphadenopathy (P<0.05). The further comparison between every two groups showed that maximum diameter larger than 5.9 cm, heterogeneous density, and pericardial or pleural effusion were more common in grade III than in grades I and II.

**Table 3:** Association between clinical and CT features and artery invasion grades in thymic epithelial tumors.

Variables	Grade I (< 25%) (n = 67)	Grade II (25 - 49%) (n = 42)	Grade III (≥ 50%) (n = 13)	*P*
Gender, n (%)				< 0.001^a,b^
Male	26 (38.8)	29 (69.0)	12 (92.3)	
Female	41 (61.2)	13 (31.0)	1 (7.7)	
Age [years, n (%)]				0.235
≤ 50	26 (38.8)	21 (50.0)	8 (61.5)	
> 50	41 (61.2)	21 (50.0)	5 (38.5)	
Myasthenia gravis, n (%)				0.263
Yes	18 (26.9)	17 (40.5)	3 (23.1)	
No	49 (73.1)	25 (59.5)	10 (76.9)	
Location, n (%)				0.055
Off-midline	44 (65.7)	35 (83.3)	7 (53.8)	
Midline	23 (34.3)	7 (16.7)	6 (46.2)	
Maximum diameter [cm, n (%)]				< 0.001^a,b,c^
≤ 5.9	55 (82.1)	15 (35.7)	0 (0.0)	
> 5.9	12 (17.9)	27 (64.3)	13 (100.0)	
Contour, n (%)				< 0.001^a,b^
Smooth	28 (41.8)	4 (9.5)	0 (0.0)	
Lobulated	39 (58.2)	38 (90.5)	13 (100.0)	
Homogeneity, n (%)				< 0.001^a,b,c^
Almost homogenous	42 (62.7)	15 (35.7)	0 (0.0)	
Heterogeneous	25 (37.3)	27 (64.3)	13 (100.0)	
Enhancement degree, n (%)				0.004^a^
Mild to moderate	50 (74.6)	40 (95.2)	13 (100.0)	
Strong	17 (25.4)	2 (4.8)	0 (0.0)	
Pericardial or pleural invasion, n (%)				< 0.001^a,b^
Yes	19 (28.4)	38 (90.5)	13 (100.0)	
No	48 (71.6)	4 (9.5)	0 (0.0)	
Lung invasion, n (%)				< 0.001^a,b^
Yes	7 (10.4)	27 (64.3)	12 (92.3)	
No	60 (89.6)	15 (35.7)	1 (7.7)	
Pericardial or pleural effusion, n (%)				< 0.001^a,b,c^
Yes	6 (9.0)	19 (45.2)	11 (84.6)	
No	61 (91.0)	23 (54.8)	2 (15.4)	
Lymphadenopathy, n (%)				< 0.001^b^
Yes	4 (6.0)	8 (19.0)	7 (53.8)	
No	63 (94.0)	34 (81.0)	6 (46.2)	

Comparisons among groups using Pearson chi-square tests or ^*^Fisher’s exact tests. p < 0.05 indicates a statistically significant difference among all groups, and adjusted p < 0.0167 (0.05/3, Bonferroni method) indicated a significant difference between the two groups. ^a^ Represents significant differences between grades I and II. ^b^ Represents significant differences between grades I and III. ^c^ Represents significant differences between grades II and III.

Patients were divided into two groups according to the invasion degree of the SVC or innominate vein. Factors associated with greater than or equal to 50% venous invasion included male patients, tumors with a maximum diameter larger than 5.9 cm, lobulated contours, heterogeneous density, pericardial or pleural invasion, adjacent lung invasion, pericardial or pleural effusion, and mediastinal lymphadenopathy (P < 0.05) (**Table 4**). Age, myasthenia gravis, tumor location, and degree of enhancement were not statistically different among the two groups (P > 0.05).

**Table 4:** Association between clinical and CT features and vein invasion grades in thymic epithelial tumors.

Variables	Grade I (< 50%) (n = 100)	Grade II (≥ 50%) (n = 22)	*P*
Gender, n (%)			0.020
Male	50 (50.0)	17 (77.3)	
Female	50 (50.0)	5 (22.7)	
Age [years, n (%)]			0.053
≤ 50	41 (41.0)	14 (63.6)	
> 50	59 (59.0)	8 (36.4)	
Myasthenia gravis, n (%)			0.346
Yes	33 (33.0)	5 (22.7)	
No	67 (67.0)	17 (77.3)	
Location, n (%)			0.070
Off-midline	74 (74.0)	12 (54.5)	
Midline	26 (26.0)	10 (45.5)	
Maximum diameter [cm, n (%)]			< 0.001
≤ 5.9	69 (69.0)	1 (4.5)	
> 5.9	31 (31.0)	21 (95.5)	
Contour, n (%)			0.002
Smooth	32 (32.0)	0 (0.0)	
Lobulated	68 (68.0)	22 (100.0)	
Homogeneity, n (%)			0.001
Almost homogenous	54 (54.0)	3 (13.6)	
Heterogeneous	46 (46.0)	19 (86.4)	
Enhancement degree, n (%)			0.192^*^
Mild to moderate	82 (82.0)	21 (95.5)	
Strong	18 (18.0)	1 (4.5)	
Pericardial or pleural invasion, n (%)			< 0.001
Yes	50 (50.0)	20 (90.9)	
No	50 (50.0)	2 (9.1)	
Lung invasion, n (%)			< 0.001
Yes	28 (28.0)	18 (81.8)	
No	72 (72.0)	4 (18.2)	
Pericardial or pleural effusion, n (%)			< 0.001
Yes	19 (19.0)	17 (77.3)	
No	81 (81.0)	5 (22.7)	
Lymphadenopathy, n (%)			0.007^*^
Yes	11 (11.0)	8 (36.4)	
No	89 (89.0)	14 (63.6)	

Comparisons among groups using Pearson chi-square tests or *Fisher’s exact tests. p < 0.05 indicates a statistically significant difference among groups.

### Multivariable analysis to predict the grades of major mediastinal vascular invasion in TETs

All indicators, including clinical and CT features, were included in the multivariable logistic regression analysis (**Table 5**). For predicting 25% invasion of the aorta or pulmonary artery and its branches, the results showed that only the male patients (β = 1.549; odds ratio, 4.824; P = 0.007) and the pericardial or pleural invasion (β = 2.209; odds ratio, 9.110; P = 0.001) were independent predictors. As for 50% invasion of aorta or pulmonary artery and its branches, the midline location (β = 2.504; odds ratio, 12.234; P = 0.01) and mediastinal lymphadenopathy (β = 2.490; odds ratio, 12.06; P = 0.008) were independent predictors. Risk factors associated with ≥50% invasion of the SVC or innominate vein included midline location (β = 2.303; odds ratio, 10.0; P = 0.008), maximum tumor diameter larger than 5.9 cm (β = 4.038; odds ratio, 56.739; P = 0.001), and pericardial or pleural effusion (β = 1.460; odds ratio, 4.306; P = 0.033).

**Table 5:** Multivariable logistic regression model for predicting the defined grades of major mediastinal artery or vein invasion in thymic epithelial tumors.

Variables	β	SE	OR (95% CI)	*R^2^*	*P*
Aorta or pulmonary artery and its branches					
25% invasion				0.711	
Male	1.549	0.578	4.824 (1.553-14.987)		0.007
Pericardial or pleural invasion	2.209	0.668	9.110 (2.459-33.749)		0.001
50% invasion				0.614	
Midline location	2.504	0.977	12.234 (1.801-83.095)		0.010
Lymphadenopathy	2.490	0.937	12.060 (1.922-75.685)		0.008
SVC or innominate vein					
50% invasion				0.562	
Midline location	2.303	0.864	10.000 (1.840-54.350)		0.008
Maximum diameter > 5.9 cm	4.038	1.256	56.739 (4.843-664.692)		0.001
Pericardial or pleural effusion	1.460	0.686	4.306 (1.122-16.519)		0.033

SE, standard error; CI, confidence interval; OR, odds ratio; SVC, superior vena cava.

### Efficacy analysis of multivariable logistic regression model and univariate features

Based on the predictive probability values derived from the logistic regression model and univariate features for predicting 50% of great mediastinal vascular invasion in TET patients, ROC analysis was performed. The multivariate model achieved a higher diagnostic efficacy with an AUC of 0.944, 84.6% sensitivity, and 91.7% specificity for predicting 50% of artery invasion, and an AUC of 0.913, 81.8% sensitivity and 86.0% specificity for predicting 50% of venous invasion in TET patients (**Table 6**).

**Table 6:** Efficacy analysis of multivariable logistic regression model and univariate features in predicting 50% of major mediastinal artery and venous invasion in thymic epithelial tumors.

Variables	50% artery invasion			50% venous invasion		
	AUC (95% CI)	Se	Sp	AUC (95% CI)	Se	Sp
Multivariate model	0.944 (0.899-0.988)	84.6	91.7	0.913 (0.850 - 0.976)	81.8	86.0
Gender	0.709 (0.586-0.832)	92.3	49.5	0.636 (0.514 - 0.758)	77.3	50.0
Maximum diameter	0.821 (0.741-0.901)	100.0	64.2	0.822 (0.741 - 0.903)	95.5	69.0
Contour	0.647 (0.519-0.775)	100.0	29.4	0.660 (0.555 - 0.765)	100.0	32.0
Homogeneity	0.761 (0.665-0.858)	100.0	52.3	0.702 (0.592 - 0.811)	86.4	54.0
Enhancement degree	0.587 (0.443-0.731)	100.0	17.4	-	-	-
Peritumoral fat infiltration	0.633 (0.501-0.765)	100.0	26.6	0.645 (0.537 - 0.753)	100.0	29.0
Pericardial or pleural invasion	0.739 (0.635-0.842)	100.0	47.7	0.705 (0.599 - 0.810)	90.9	50.0
Lung invasion	0.806 (0.700-0.911)	92.3	68.8	0.769 (0.662 - 0.876)	81.8	72.0
Pericardial or pleura effusion	0.808 (0.685-0.932)	84.6	77.1	0.791 (0.680 - 0.902)	77.3	81.0
Lymphadenopathy	0.714 (0.543-0.885)	53.8	89.0	0.627 (0.486 - 0.767)	36.4	89.0

AUC, area under curve; CI, confidence interval; Se, sensitivity; Sp, specificity.

According to the ROC analyses, some univariate features demonstrated different predictive efficacy for predicting 50% artery invasion in TET patients, including gender, tumor maximum diameter, contour, homogeneity, enhancement degree, pericardial or pleural invasion, lung invasion, pericardial or pleural effusion, and lymphadenopathy. Similarly, in addition to the enhancement degree, all of the above features were also helpful in predicting 50% venous invasion, with varying degrees of predictive efficacy (**Table 6**).

### Inter-reader agreement analysis for major mediastinal vascular invasion

Based on Cohen’s kappa coefficient, the consistency between the two readers was good in judging the invasion degree for the aorta or pulmonary artery and its branches, and SVC or innominate vein; Cohen’s kappa coefficient was 0.66 and 0.70, respectively.

## Discussion

The invasion status of the major mediastinal vessels is critical to the decisions of the treatment plan and surgical approach in patients with TET (15, 21–24). In the current study, major mediastinal vessel invasion characteristics in TETs were evaluated based on enhanced CT images, and risk factors for aortic or pulmonary artery and SVC or innominate vein abutment, including clinical and CT features, were analyzed. The results demonstrated significant differences in the abutment rate of major mediastinal vessels among different histologic types, MK and TNM stages in TETs. In addition, several CT features could be used as independent predictors of ≥50% artery or venous abutment. Prediction models based on these predictors yielded high efficacy, indicating the possibility of incomplete resection, which may be helpful in the formulation of surgical protocols and screening of patients who benefit from neoadjuvant chemotherapy.

The types and stages of TETs reflect their invasiveness, which is closely associated with the invasion degree of major mediastinal vessels. According to the current and previous study results, the incidence and degree of vascular invasion in high-risk thymoma and thymic carcinoma were higher than those of low-risk thymoma (5, 25–28). Similar to the previous studies (26–28), the current results revealed that advanced-stage tumors had a higher vascular invasion degree. All the cases with ≥50% abutment of aortic, pulmonary artery, or pulmonary vein were advanced-stage tumors, while patients with ≥50% abutment of SVC or innominate vein were also mostly advanced-stage tumors. In addition, the most commonly invaded vessels are the SVC or innominate vein, followed by the aorta and its branches, the pulmonary artery, and the pulmonary vein, which may be related to the anatomical characteristics of the abutment vessel, as well as their proximity to the tumors.

Compared to MK staging, the new TNM staging system has the advantage of evaluating TET invasiveness in more detail, aiming to evaluate tumors in clinical settings and provide more information for formalizing resectability (29, 30). In the current study, whether it is an artery or a vein, ≥ 50% of vascular abutment was mainly seen in TNM stages III and IV. However, there was no significant statistical significance between the III and IV stages of vascular abutment with ≥ 50%. This may be related to the fact that all T4 cases in this group have lymph node, pericardial, pleural, or hematogenous metastases, which are classified as stage IV.

Different histologic types of TET have different vascular invasion characteristics, resulting in different surgical procedures and prognoses (22–24). Several clinical risk factors have been proposed to evaluate the histological subtypes of TET patients (31), which be related to the invasion of major mediastinal vessels. This study confirmed the higher incidence of high-grade vascular invasion in male patients, which may be attributed to the higher incidence of thymic carcinoma in males (16, 32). Similar to the result of no significant difference in age among different types of TETs, there was no significant difference in age among patients with different vascular invasion grades (16, 31). Although myasthenia gravis is particularly common in types AB, B1, and B2 thymoma (16), no significant difference was found among TET patients with different grades of vascular invasion.

CT is the preferred imaging modality for evaluating the tumor types and stages in TET patients (31). Several CT features of TET, including maximum diameter, contour, homogeneity, enhancement degree, pericardial or pleural invasion or effusion, lung invasion, and lymphadenopathy, are associated with the major mediastinal vessel invasion grade. Although there was no statistical difference in tumor location among patients with different grades of arterial or venous invasion, vascular invasion was more common in tumors with off-midline locations, which may be related to the lateral growing nature of thymomas (27, 33). In addition, the proportion of midline location in high-grade vascular invasion is higher than that in low-grade vascular invasion based on the results of this study, suggesting that tumors in midline locations have a higher probability of 50% vascular invasion. Tumor size significantly influences the choice of surgical approach and is a strong predictor of prognosis in TETs (12, 34, 35). Similarly, the results of this study showed that tumors with a maximum diameter larger than 5.9 cm have higher vascular invasion grades in both arterial and venous vessels.

According to the current results, high-grade vascular invasion occurred mostly in lobulated and heterogeneous tumors, which could be associated with its high aggressiveness and growth pattern (16, 28, 31). Furthermore, high-grade vascular invasion is rare in strong contrast-enhanced cases, which may be related to the fact that TETs with strong contrast enhancement are mainly seen in type AB thymoma (16, 36). Mediastinal lymphadenopathy is primarily seen in thymic carcinoma (25, 31, 37–39), with a higher incidence of vascular invasion. In addition, this study found that the incidence of high-grade vascular invasion in TET patients with pericardial or pleural and lung invasion was significantly increased. Ultimately, CT features of tumors are closely related to tumor invasiveness, reflecting tumor growth patterns and vascular invasion grade.

It is critically important to accurately identify the extent of vascular invasion before surgery (13). Therefore, based on key clinical and CT features of TETs, this study conducted a multivariable logistic regression analysis to predict 25% and 50% invasion of artery vessels, and 50% venous vessel invasion, respectively. The results revealed that midline location and lymphadenopathy are two effective independent predictors in identifying 50% invasion of the aorta or pulmonary artery and its branches. Correspondingly, midline location, maximum diameter larger than 5.9 cm, and pericardial or pleural effusion are independent factors in predicting 50% invasion of SVC and innominate vein.

ROC analysis confirmed that the logistic model obtained higher efficacy in predicting 50% of major mediastinal vascular invasion compared with the single-factor parameters, with an AUC of 0.944 and 0.913 for artery and venous vessel invasion, respectively. Therefore, the multivariate regression model is helpful in predicting the resectability of major mediastinal vessels in TETs.

An inter-reader agreement analysis was performed to evaluate the reliability in judging the extent of mediastinal vascular invasion between two independent radiologists in this study. The results showed good consistency between the two readers, with a kappa coefficient of 0.66 and 0.70, in evaluating the arterial and venous system invasion.

## Limitations

There were several limitations of this study. Firstly, due to the nature of the retrospective study, the vascular invasion extent was evaluated using CT imaging but not confirmed by surgery, which may introduce bias in ROC analysis. Secondly, three CT scanners with different scanning protocols were used in this study. However, we believe that these parameters will not strongly affect the evaluation of CT features in TETs. Thirdly, the sample size is relatively small, and validation testing of the logistic model has not yet been conducted. Therefore, further prospective studies are warranted to collect surgical information on more TET patients to clarify this research issue.

## Conclusion

In conclusion, several CT features can be used as independent predictors of ≥50% artery or venous abutment for TET patients. The multivariate logistic model based on the key CT features is valuable for predicting the invasion degree of major mediastinal vascular in TETs, which may aid clinical decision-making in patients facing surgery or neoadjuvant chemotherapy.

## Data availability statement

The raw data are not publicly available due to them containing information that could compromise research participant privacy/consent. Requests to access the datasets should be directed to G-BC, cgbtd@126.com.

## Ethics statement

The studies involving humans were approved by Tangdu Hospital, Fourth Military Medical University. The studies were conducted in accordance with the local legislation and institutional requirements. The ethics committee/institutional review board waived the requirement of written informed consent for participation from the participants or the participants’ legal guardians/next of kin because this study is a retrospective study.

## Author contributions

G-BC and Y-CH conceived the study. Y-HM, W-QY and JZ participated in the study design. Y-HM, W-QY, JZ, J-TL, X-LF, S-MW and GY performed the data acquisition, Y-HM and Y-CH participated in the statistical analyses. All authors participated in the data interpretation. Y-HM drafted the first version of the report. All authors contributed to the article and approved the submitted version.
