# Supplementary material for: Risk factor analysis for major mediastinal vessel invasion in thymic epithelial tumors based on multi-slice CT Imaging
Source: Front Oncol. 2023 Sep 11;13:1239419. doi: 10.3389/fonc.2023.1239419 (PMC10518454; doi:10.3389/fonc.2023.1239419)
Supplement: Supplementary file 1 [file Table_1.docx]

**Supplemental Table 1** **|** TNM staging of 122 patients with thymic epithelial tumors

|  |  | value |
| --- | --- | --- |
| TNM stage, n (%) |  |  |
| T |  |  |
| 1 |  | 76 (62.3) |
| 2 |  | 7 (5.7) |
| 3 |  | 29 (23.8) |
| 4 |  | 10(8.2) |
| N |  |  |
| 0 |  | 104(85.2) |
| 1 |  | 2(1.6) |
| 2 |  | 16(13.1) |
| M |  |  |
| 0 |  | 95(77.9) |
| 1a |  | 16(13.1) |
| 1b |  | 11(9.0) |
